# Determinants for perinatal adverse outcomes among pregnant women with preterm premature rupture of membrane: A prospective cohort study

**DOI:** 10.3389/frph.2022.1052827

**Published:** 2022-12-15

**Authors:** Tariku Abewa Abebe, Dawit Desalegn Nima, Yitbarek Fantahun Mariye, Abebaye Aragaw Leminie

**Affiliations:** ^1^ Department of Obstetrics and Gynecology, School of Medicine, College of Health Sciences, Addis Ababa University, Addis Ababa, Ethiopia; ^2^ Department of Medical Physiology, School of Medicine, College of Health Sciences, Addis Ababa University, Addis Ababa, Ethiopia

**Keywords:** APGAR, outcomes, preterm, pregnancy, rupture, NICU

## Abstract

**Background:**

One of the most critical functions of the fetal membranes is to remain intact until the onset of labor to maintain the protective intrauterine fluid environment. In most pregnancies, spontaneous rupture usually occurs near the end of the first stage of labor. Preterm premature membrane rupture (PROM) occurs when the fetal membrane ruptures before 37 weeks of pregnancy, and it contributes to adverse maternal, fetal, and neonatal outcomes. Therefore, this study aimed to determine the association of determinant factors with adverse perinatal outcomes.

**Methods:**

A prospective cohort study was conducted on pregnant women with preterm premature membrane rupture (*n* = 160) attending the teaching hospitals at Addis Ababa University. Socio-demographic and obstetric risk factors with adverse perinatal outcomes include the 5th minute Apgar score, neonatal intensive care unit (NICU) admission, early-onset neonatal sepsis (EONS), respiratory distress syndrome (RDS), perinatal mortality, Chorioamnionitis, and placental abruption were assessed. SPSS version 24, *t*-test, *χ*^2^ test, and logistic regression analysis were used. *P*-values <0.25 in the bivariate and *p* < 0.05 in the multiple logistic regression were considered statistically significant.

**Results:**

The preterm (PROM) rate was 2.2% with perinatal mortality rate of 206/1,000. Gestational age (GA) at delivery was the determinate for low Apgar score at the 5th minute (AOR: 7.23; 95% CI, 1.10, 47.6; *p* = 0.04). Unable to use steroid (AOR: 8.23; 95% CI, 1.83, 37.0; *p* = 0.000), GA at membrane rupture (AOR: 4.61; 95% CI, 1.98, 31.8; *p* = 0.000) and delivery (AOR: 4.32; 95% CI, 1.99, 30.9; *p* = 0.000) were determinates for NICU admission. EONS was significantly affected by GA at membrane rupture (AOR: 5.9; 95% CI, 1.01, 37.0; *p* = 0.04). Placental abruption was significantly affected by GA at delivery (AOR: 7.52; 95% CI, 1.15, 48.96; *p* = 0.04).

**Conclusion:**

GA at membrane rupture and delivery was the most critical predictors of adverse perinatal outcomes. Local guidelines on the approach and preterm PROM outcome management need to be prepared.

## Introduction

### Background of the study

One of the most critical functions of the fetal membranes is to remain intact until the onset of labor to maintain the protective intrauterine fluid environment (1). In about 70% of pregnancies without interventions, their spontaneous rupture usually occurs near the end of the first stage of labor. Spontaneous membrane rupture occurs physiologically due to the progressive weakening of the membranes with advancing gestational age (GA). Premature rupture is also common among pregnant women with previous preterm premature rupture of membrane (PROM), previous preterm delivery, history of cigarette smoking, bleeding during pregnancy, and genitourinary infections (2, 3). It is one of the most common pregnancy complications (4).

Membrane rupturing after 37 weeks of gestation is term PROM and is preterm if the membrane ruptures before 37 weeks of pregnancy (1, 5, 6). Preterm PROM results in adverse perinatal outcomes (1, 4, 6). Preterm birth, early-onset neonatal sepsis (EONS), admission to the neonatal intensive care unit (ICU), stillbirth, clinical Chorioamnionitis, abruption placentae, intraventricular hemorrhage, RDS, fetal death, and sepsis are some of the maternal, fetal and neonatal adverse outcomes (7–10). Fifteen million babies are born preterm worldwide, and 1.1 million infants die due to preterm complications each year (11).

Few studies indicated that neonatal complications are inversely related to the GA at membrane rupture and delivery (8, 10, 12). However, no studies have been conducted on the strong predictor factors for adverse perinatal outcomes prospectively. Therefore, this study aimed to identify determinants affecting the adverse perinatal outcomes among women with preterm PROM. Identifying the risk factors for fetal and neonatal outcomes among pregnant women with preterm PROM is essential to improve neonatal outcomes.

## Subjects and methods

### Study area and period

The study was conducted in Black lion, Zewuditu, and Gandhi Memorial teaching hospitals in Addis Ababa. These hospitals have neonatal intensive care units for the admission and management of neonates. The research data were collected for five months, from April to August 2018, from three hospitals.

### Study design and population

The design we used in this study was an institutional prospective cohort study. In this prospective cohort study, initially, pregnant women who had complications with preterm PROM cases were identified by trained data collectors (nurses) using an antenatal-log book and followed until they gave birth. In addition, women who gave alive-born were followed through the early neonatal period for one week.

Thus, pregnant women who had preterm PROM-induced complications and gave birth in the three teaching hospitals were included in the study. Women with preterm PROM, singleton pregnancy between 28 and 37 weeks of GA, and who gave written informed consent were recruited. Pregnant women with known diseases such as pregnancy-induced hypertension, gestational diabetes mellitus, and cardiac and kidney problems were excluded from this study.

### Sample size and sampling technique

The sample size in this study was determined using a single population proportion formula, *n* = Z^2^ * pq/e^2^. Where N is the sample size, Z is the Z score (1.96 at 95% CI), *p* is the prevalence of prenatal mortality rate attributed to preterm PROM and was reported to be 107/1,000 (8), q (1-*p*) is the level of precision and e is the marginal error (5%). With a 10% non-response rate, the final sample size in this study was 160. A convenience sampling technique was used to recruit the study participants. Those women whose pregnancies were complicated by preterm PROM, delivered in the three teaching hospitals, and fulfilled the inclusion criteria were enrolled in the study until the calculated sample size was completed.

### Study variables

Age, ethnicity, religion, occupation, marital status, educational status, family income, address parity, antenatal care booking status, site of antenatal care, and GA at membrane rupture were independent variables in this study. Fetal presentation, latency period, duration of labor, use of prophylactic antibiotics, and corticosteroids were also the independent variables.

The outcome variables in this study were perinatal mortality rate, Apgar score at the 5th minute, need for neonatal intensive care unit admission, and EONS. Fetal RDS, clinical Chorioamnionitis, and abruption placentae were also the outcome variables in this study.

### Data collection

Cases were identified from the delivery logbook every day. The required data were compiled by trained data collectors using structured data collection tools and reviewing charts of women who gave birth and had preterm PROM. The neonates were followed through the early neonatal period. At delivery, detailed histories were taken using structured questionnaires. GA at membrane rupture and delivery, latency period, and duration of labor recorded. GA was determined using obstetric estimation.

Type, duration, and use of antibiotics and steroids before childbirth (antepartum) and mode of delivery were recorded. Soon after delivery, birth weight, sex, and Apgar score at the 5th minute were also recorded. Neonates were referred to the neonatal intensive care unit, and admission was determined.

Death, stillbirth, 8th day of stay in the neonates' intensive care unit, presence or absence of EONS, and RDS were evaluated by trained data collectors, and two BSc nurses from each hospital, by reviewing their charts. Chorioamnionitis and placental abruption were obtained from the maternal chart. Clinical Chorioamnionitis and placental abruption, in this study, were diagnosed solely based on clinical signs since access to uncontaminated amniotic fluid or placenta for culture is invasive and usually avoided (13, 14).

### Data analysis

Data were entered using SPSS version 24.00. The logistic regression model was used to estimate the association of maternal exposure and birth outcomes with adverse perinatal outcomes. Independent T and Chi-square tests were also used. Parity, antenatal care status, GA at membrane rupture and delivery, latency period, duration of labor, antibiotics, and corticosteroid uses were identified *a priori*. Results were presented as mean ± standard deviation (SD). A *p*-value of less than 0.25 at bivariate and 0.05 during multivariate logistic regression at 95% CI was considered statistical significance.

### Ethical consideration

The study was conducted after approval, and ethical clearance was obtained from the Ethical and Review Committee of the Department of Obstetrics and Gynecology. The Institutional Review Board of the college also approved the study. Data were collected after the principal investigator explained the study, and informed consent was obtained from the participants.

## Results

### Socio-demographic characteristics

Out of 160 deliveries with preterm PROM, 59 (36.9%), 53 (33.1%), and 48 (30%) were at Black-lion, Zewditu, and Gandhi Memorial Hospitals, respectively. A maximum number (*n* = 99, 62%) of preterm PROM was observed in the age between 25 and 34 years, while the mean age (year) of women was 26.7 ± 4.60. Most of the participants (*n* = 70, 44%) were in the middle-income category. Among the study participants, 27 (17%) graduated from tertiary education, 68 (48%) from high school dropouts, 54 (34%) women had primary education, and 6 (4%) pregnant women had no formal education and were unable to read and write. In comparison, 5(3%) were able to read and write (Table 1).

**Table 1:** Socio-demographic characteristics among pregnant women with preterm premature rupture of membrane.

Variable	Category	*N* (%)	Variable	Category	*N* (%)
Age (year)	<20	8 (5)	Income	Low	37 (23)
	20–24	42 (26)		Middle	70 (44)
	25–34	99 (62)		High	53 (33)
	≥35	11 (7)	Education	Unable to read and write	6 (4)
Ethnicity	Amhara	49 (31)		Able to read and write	5 (3)
	Oromo	26 (16)		Primary education	54 (34)
	Gurage	52 (33)		High school	68 (43)
	Tigre	15 (9)		Tertiary education	27 (17)
	Others	18 (11)			

#### Determinants for adverse perinatal outcomes

In this study, the total number of women who gave birth during the data collection period was 7,235. Of these, 160 women had preterm PROM (rate of 2.2%) with the GA range between 28 and 36 weeks +6 days. Among 160 deliveries, neonates with the low Apgar score at the 5th minute were 41 (26%). In the bivariate analysis, GA at membrane rupture (*p* < 0.01) and delivery (*p* < 0.001) was significantly associated with the low Apgar score at the 5th minute. Membrane rupture and delivery at GA between 28 and 33 weeks were 4.7 and 7.7 times higher than membrane rupture and delivery within weeks between 34 and 36 to have a low Apgar score. However, parity, birth weight, delivery mood, antibiotics, and steroids use, latency period, and antenatal care visit did not affect the Apgar score (Table 2).

**Table 2:** Factors determining the Low 5th Minute Apgar score among pregnant women with preterm premature rupture of membrane.

Variable	Category	5th minute As		OR (95% CI)
		<7	≥7	
		*N* (%)	*N* (%)	
Parity	Nulliparous	25 (30.1)	58 (69.9)	1.6 (0.8, 3.4)
	Multiparous	16 (20.8)	61 (79.2)	1.0
Delivery mode	Cesarean	3 (11.1)	24 (88.9)	0.3 (0.1,1.1)
	Vaginal	38 (28.6)	95 (71.4)	1.0
Use of Prophylactic Abs	Yes	33 (23.4)	108 (76.6)	0.4 (0.2,1.1)
	No	8 (4.1)	11 (57.9)	1.0
Use of steroids	Yes	8 (33.3)	16 (66.7)	1.6 (0.6,3.9)
	No	33 (24.3)	103 (75.7)	1.0
GA at ROM (week)	28–33 + 6	18 (51.4)	17 (48.6)	4.7 (2.1, 10.5)**
	34–36 + 6	23 (18.4)	102 (81.6)	1.0
GA at delivery (week)	28–33 + 6	17 (63.0)	10 (37.0)	7.7 (3.2, 18.9)***
	34–36 + 6	24 (18)	109 (82)	1.0
Latency	<72 h	33 (27.7)	86 (72.3)	1.6 (0.7, 3.8)
	≥72 h	8 (19.5)	33 (80.5)	1.0
Birth weight	Low	8 (14.3)	48 (85.7)	1.9 (0.6,6.1)
	Normal	6 (7.8)	71 (92.2)	1.0
Maternal age (year)	<35	40 (26.7)	110 (73.3)	3.3 (0.4,26.7)
	≥35	1 (10.0)	9 (90.0)	1.0
Antenatal care	Yes	40 (25.3)	118 (34.7)	1.0
	No	1 (50.0)	1 (50.0)	0.3 (0.0,5.6)

As, Apgar score; Abs, Antibiotics; GA, Gestational age; ROM, Rupture of Membrane. ** & *** indicate statistically significant difference at *p* < 0.01 and *p* < 0.001, respectively.

Among 133 alive born neonates, 79 (59%) were admitted to NICU and the most diagnoses at admission were EONS (*n* = 55, 41%) followed by RDS (*n* = 40, 30%). Among neonates admitted to the neonatal ICU, 53.6% did not take steroids for lung maturation. Birth weight (*p* < 0.001), GA at membrane rupture (*p* < 0.001) and delivery (*p* < 0.05), and use of steroids (*p* < 0.01) were significantly associated with the NICU admission. Neonates born with low weight were 5 times higher than those born with normal body weight to be admitted to the NICU (Table 3).

**Table 3:** Factors determining NICU admission among pregnant women with preterm premature rupture of membrane.

Variable	Category	NICU admission		OR (95% CI)
		Yes	No	
		*N* (%)	*N* (%)	
Parity	Nulliparous	40 (58.8)	28 (41.2)	0.9 (0.5,1.9)
	Multiparous	39 (60.0)	26 (40.0)	1.0
Mode of delivery	Cesarean	18 (69.2)	8 (30.8)	1.7 (0.7,4.2)
	Vaginal	61 (57.0)	46 (43.0)	1.00
Use of Prophylactic Abs	Yes	67 (56.8)	51 (43.6)	0.3 (0.1,1. 2)
	No	12 (80.0)	3 (20.0)	1.0
Use of steroids	Yes	19 (24.1)	2 (9.5)	1.0
	No	60 (53.6)	52 (46.3)	8.2 (1.8,37.0)**
GA at ROM (week)	28–33 + 6	20 (100.0)	0 (0.0)	1.9 (1.6,2.9)***
	34–36 + 6	59 (52.2)	54 (47.8)	1.0
GA at delivery (week)	28–33 + 6	13 (100.0)	0 (0.0)	1.8 (1.6,2.1)*
	34–36 + 6	66 (55.0)	54 (45)	1.0
Latency	<72 h	52 (54.7)	43 (45.3)	0.5 (0.2,1.1)
	≥72 h	27 (71.1)	11 (28.9)	1.0
Birth weight (gm)	Low	45 (80.4)	11 (19.6)	5.2 (2.3,11.5)***
	Normal	34 (44.2)	43 (55.8)	1.0

NICU, Neonatal intensive care unit; Abs, antibiotics; GA, Gestational Age; ROM, Rupture of Membrane.

*, ** & *** indicate statistically significant difference at *p* < 0.05, *p* < 0.01 and *p* < 0.001, respectively.

On the other hand, among 133 alive-born neonates, 41.4% (*n* = 55) had EONS of which 9 (6.8%) were cesarean deliveries. The rate of development of RDS among alive-born neonates was 30% (*n* = 40), from which 7.5% (*n* = 10) were delivered by cesarean section. In this study, 133 (83%) were born alive, while 19 (12%) and 8 (5%) were antepartum and intrapartum death, respectively. The total fetal deaths before and during labor were 27 (17%). Among neonates born alive, 6 (5%) died in the first week after delivery. In this study, the perinatal mortality rate was 206 per 1,000 births. Out of 160 women with preterm PROM, 12 (8%) women had clinical Chorioamnionitis.

EONS (*p* < 0.01), RDS (*p* < 0.05), and perinatal mortality (*p* < 0.001) were significantly affected by GA at PROM. GA at PROM at less than 34 weeks showed 5.5, 2.8, and 6.4 times higher risk of developing EONS, RDS, and to have peritoneal mortality respectively when compared with GA at PROM at ≥34 weeks (Tables 4–6). GA at delivery at less than 34 weeks showed a 3.1 and 9.9 times higher risk of developing RDS and having peritoneal mortality respectively when compared with GA at delivery at ≥34 weeks (Tables 5, 6).

**Table 4:** Factors determining the development of EONS among pregnant women with preterm premature rupture of membrane.

Variable	Category	EONS		OR (95% CI)
		Yes	No	
		*N* (%)	*N* (%)	
Delivery mode	Cesarean	9 (34.6)	17 (65.4)	0.7 (0.3,1.7)
	Vaginal	46 (43.0)	61 (57.0)	1.0
Use of Prophylactic Abs	Yes	50 (42.4)	68 (57.6)	1.5 (0.4,4.6)
	No	5 (33.3)	10 (66.7)	1.00
Use of steroids	Yes	14 (58.3)	10 (41.7)	3.5 (1.3,9.3)*
	No	41 (30.1)	95 (69.9)	1.0
GA at ROM (week)	28–33 + 6	15 (75.0)	5 (25.0)	5.5 (1.9, 16.8)**
	34–36 + 6	40 (35.4)	73 (64.6)	1.0
GA at delivery (week)	28–33 + 6	9 (69.2)	4 (30.8)	3.6 (1. 1, 12.4)
	34–36 + 6	46 (38.3)	74 (61.7)	1.0
Latency	<72 h	36 (37.9)	59 (62.1)	0.6 (0.3,1.3)
	≥72 h	19 (50.0)	19 (50.0)	1.0
Birth weight (gm)	Low	32 (57.1)	24 (42.9)	3.13 (1.5,6.4)**
	Normal	23 (29.9)	54 (70.1)	1.0
Labor duration (hours)	<24	46 (31.3)	101 (68.7)	1.0
	≥24	9 (69.2)	4 (30.8)	4.9 (0.1,0.7)**
Chorioamnionitis	Yes	7 (58.3)	5 (41.7)	2.9 (0.9,9.7)
	No	48 (32.4)	100 (67.6)	1.0

EONS, Early-onset neonatal sepsis; Abs, antibiotics; GA, Gestational age; ROM, rupture of membrane.

*, ** & *** indicate statistically significant difference at *p* < 0.05, *p* < 0.01, *p* < 0.001, respectively.

**Table 5:** Factors determining the development of RDS among pregnant women with preterm premature rupture of membrane.

Variable	Category	RDS		OR (95% CI)
		Yes	No	
		*N* (%)	*N* (%)	
Delivery mode	Cesarean	10 (38.5)	16 (61.5)	1.6 (0.7,3.9)
	Vaginal	30 (28.0)	77 (72.0)	1.0
Use of Prophylactic Abs	Yes	33 (28.0)	85 (72.0)	0.4 (0.2,1.3)
	No	7 (46.7)	8 (53.3)	1.0
Use of steroids	Yes	9 (42.9)	12 (57.1)	1.9 (0.8,5.1)
	No	31 (27.7)	81 (72.3)	1.0
GA at ROM (week)	28–33 + 6	10 (50.0)	10 (50.0)	2.8 (1.0, 7.3)*
	34–36 + 6	30 (26.5)	83 (73.5)	1.0
GA at delivery (week)	28–33 + 6	7 (53.8)	6 (45.2)	3.1 (1.9,9.8)**
	34–36 + 6	33 (27.5)	87 (72.5)	1.0
Latency period (hours)	<72	27 (28.4)	68 (71.6)	0.8 (0.3,1.7)
	≥72	13 (34.2)	25 (65.8)	1.0
Birth weight (gm)	Low	23 (41.1)	33 (58.9)	2.5 (1.2,5.3)*
	Normal	17 (22.1)	60 (77.9)	1.0
Chorioamnionitis	Yes	6 (50.0)	6 (50.0)	3.35 (1.0,11.0)*
	No	34 (23.0)	114 (77.0)	1.0
Maternal age (year)	<35	36 (24.0)	114 (76.0)	1.0
	≥35	4 (40.0)	6 (60.0)	0.5 (0.1,1.8)

RDS, respiratory distress syndrome; GA, Gestational age; Abs, Antibiotics; ROM, rupture of membrane.

* & **, indicate statistically significant difference at *p* < 0.05 and *p* < 0.01, respectively.

**Table 6:** Factors determining perinatal mortality among pregnant women with preterm premature rupture of membrane.

Variable	Category	Perinatal mortality		OR (95% CI)
		Yes	No	
		*N* (%)	*N* (%)	
Parity	Nulliparous	19 (22.9)	64 (77.1)	1.3 (0.6,2.9)
	Multiparous	14 (18.2)	63 (81.8)	1.09
Mode of delivery	Cesarean	3 (11.1)	24 (88.9)	0.49 (0.119,1.5)
	Vaginal	30 (22.6)	103 (77.4)	1.07
Use of Prophylactic Abs	Yes	28 (19.9)	113 (80.1)	0.7 (0.2,2.1
	No	5 (26.3)	14 (73.7)	1.07
Use of steroids	Yes	5 (20.8)	19 (79.2)	1.0 (0.3,2.9)
	No	28 (20.6)	108 (79.4)	1.0
GA at ROM (week)	28–33 + 6	17 (48.6)	18 (51.4)	6.4 (2.8, 14.9)*
	34–36 + 6	16 (12.8)	109 (87.2)	1.0
GA at delivery (week)	28–33 + 6	16 (59.3)	11 (40.7)	9.9 (3.95, 24.9)*
	34–36 + 6	17 (12.8)	116 (87.2)	1.00
Latency period (hours)	>72	29 (24.4)	90 (75.6)	1.13 (0.2, 6.4)
	≥72	4 (9.8)	37 (90.2)	1.0
Birth weight (gm)	Low	25 (32.5)	52 (67.5)	4.4 (1.3,4.5)*
	Normal	8 (9.6)	75 (90.4)	1.0
RDS	Yes	5 (12.5)	35 (87.5)	1.0
	No	28 (23.3)	92 (76.7)	2.1 (0.8,5.9)
Labor duration (hours)	<24	32 (21.8)	115 (78.2)	1.4 (0.0,2.4)
	≥24	1 (7.7)	12 (92.3)	1.0

GA, Gestational Age; Abs, Antibiotics; ROM, Rupture of Membrane. *Indicates statistically significant.

Different at *p* < 0.001.

At the same time, EONS (*p* < 0.001), RDS (*p* < 0.05), and perinatal mortality (*p* < 0.001) were also significantly affected by birth weight. Neonates with low birth weight showed 3, 2.5, and 4.4 times higher risk of developing EONS, RDS, and to have peritoneal mortality respectively when compared with neonates with normal birth weight (Tables 4–6). RDS (*p* < 0.01), and perinatal mortality (*p* < 0.001) were affected by GA at delivery. The latency period (*p* < 0.01) and labor duration (*p* < 0.01) significantly affected Chorioamnionitis. Latency period ≥72 h and labor duration ≥24 h revealed 4.7 and 4.6 times higher risk for developing Chorioamnionitis than the latency period <72 h and labor duration <24 h, respectively (Table 7).

**Table 7:** Factors determining chorioamnionitis among pregnant women with preterm premature rupture of membrane.

Variable	Category	Chorioamnionitis		OR (95% CI)
		Yes	No	
		*N* (%)	*N* (%)	
GA at ROM (week)	28–33 + 6	3 (8.6)	32 (91.4)	1.2 (0.3,4.7)
	34–36 + 6	9 (7.2)	116 (92.8)	1.0
GA at Delivery (week)	28–33 + 6	0 (0.0)	27 (17.0)	1.0
	34–36 + 6	12 (8)	121 (76.0)	2.6 (1.1,1.3)
Latency period (hours)	<72	4 (3.5)	115 (95.8)	1.0
	≥72	7 (17.1)	34 (82.9)	4.7 (1.4, 15.7)**
Labor duration (hours)	<24	8 (6.6)	113 (93.4)	1.0
	≥24	3 (25.0)	9 (75.0)	4.6 (1.1, 19.7)**
Prophylactic Abs	Yes	11 (9.3)	107 (90.7)	1.5 (0.9,0.9)
	Yes	11 (8.3)	122 (91.7)	1.0
Parity	Nulliparous	6 (7.2)	77 (92.8)	1.0
	Multiparous	6 (7.8)	71 (92.2)	0.9 (0.3,2.9)
Educational status	Unable to write/read	0 (0.0)	6 (100.0)	1.0
	Able to write/read	12 (7.8)	142 (92.2)	1.0 (1.0,1.1)

GA, Gestational age; Abs, Antibiotics; ROM, rupture of membrane. * & ** indicates statically.

Significance difference at *p* < 0.05 & *p* < 0.01, respectively.

In this study, 20 (12.5%) women had a placental abruption. GA at membrane rupture in weeks less than 34 was significantly the risk for (OR: 3.59; 95% CI, 1.35–9.54, *p* < 0.05) placental abruption than membrane rupture at weeks greater than 34. Delivery in weeks less than 34 was also a significant risk for placental abruption than delivery in weeks greater than 34 (OR: 4.25; 95% CI, 1.54, 11.74, *p* < 0.01). However, latency period, labor duration, use of antibiotics, birth weight, maternal age, and parity had no significant effect on placental abruption (Table 8).

**Table 8:** Factors determining placental abruption among pregnant women with preterm premature rupture of membrane.

Variable	Category	Placental abruption		OR (95% CI)
		Yes	No	
		*N* (%)	*N* (%)	
GA at ROM (week)	28–33 + 6	9 (25.7)	26 (74.3)	3.6 (1.4,9.5)*
	34–36 + 6	11 (8.8)	114 (91.2)	1.0
GA at Delivery (week)	28–33 + 6	8 (29.6)	19 (13.6)	4.2 (1.5,11.7)**
	34–36 + 6	12 (9.0)	121 (84.4)	1.00
Latency period (hours)	<72	15 (12.6)	104 (74.3)	1.0 (0.4,3.1)
	≥72	5 (12.2)	36 (25.7)	1.0
Labor duration (hours)	<24	20 (13.6)	127 (86.4)	2.02 (0.2,0.2)
	≥24	0 (0.0)	13 (100.0)	1.0
Prophylactic Abs	Yes	17 (12.1)	124 (87.9)	1.0
	No	3 (15.8)	16 (84.2)	1.1 (1.1,1.2)
Birth weight (gm)	Low	10 (13.0)	67 (87.0)	1.0
	Normal	10 (12.5)	73 (87.5)	1.1 (0.4,2.8)
Maternal age (year)	<35	20 (13.0)	130 (86.7)	1.1 (1.0,1.1)
	≥35	0 (0.0)	10 (100.0)	1.0
Monthly income	Below average	13 (13.3)	85 (86.7)	1.1 (0.4,2.9)
	Average/above	7 (12.5)	49 (87.5)	1.0
Educational status	Unable to write /read	2 (33.3)	4 (66.7)	3.8 (0.7,22.1)
	Able to write/read	18 (11.7)	136 (88.3)	1.0
Parity	Nulliparous	11 (13.3)	77 (86.7)	1.2 (0.1,2.9
	Multiparous	9 (11.7)	68 (88.3)	1.0

GA, Gestational age; Abs, Antibiotics; ROM, rupture of membrane. * & ** indicate statistically significance difference at *p* < 0.05 & *p* < 0.01, respectively.

In multivariate logistic regression analysis, GA at delivery less than 34 weeks had 7.23 times higher risk of being born with a low Apgar score at the 5th minute (AOR: 7.23; 95% CI, 1.10, 47.6; *p* < 0.05). However, the 5th-minute Apgar score was not significantly affected by parity, mode of delivery, prophylactic antibiotics and steroids use, GA at membrane rupture, latency period, labor duration, and birth weight. NICU admission was significantly affected by unable use of steroids (AOR: 8.23; 95% CI, 1.83, 37.0; *p* < 0.001), early GA at membrane rupture (AOR: 4.61; 95% CI, 1.98, 31.8; *p* < 0.001) and delivery (AOR: 4.32; 95% CI, 1.99, 30.9; *p* < 0.001). Admission to NICU was 8.23, 4.61, and 4.3 times higher in women who failed to use steroids, had membrane ruptured, and delivered at 28–33 + 6 weeks. Contrarily, the admission was not affected by the use of antibiotics, latency period, labor duration, and birth weight.

On the other hand, GA at membrane rupture in weeks less than 34 was found to have a 5.9 higher risk of developing EONS (AOR: 5.9; 95% CI, 1.01,37.0; *p* < 0.05). However, other variables did not contribute to the development of EONS. Placental abruption was significantly affected by GA at delivery (AOR: 7.52; 95% CI, 1.15, 48.96; *p* < 0.05) (Table 9).

**Table 9:** Factors determining the major outcomes of the study among pregnant women with preterm premature rupture of membrane.

Predictor variable	Stat	Outcome variable						
		5th minute Apgar score	NICU admission	EONS	RDS	Perinatal mortality	Chor	Placental abruption
Use of Abs	Aor	0.7 (0.2,2.0)	0.3 (0.1,1.2)	0.3 (0.1,1.5)	1.9 (0.5,6.4	0.6 (0.1,2.3)	1.2 (0.3–5.3)	0.6 (0.2,2.2)
Use of Steroid	Aor	0.9 (0.3,3.6)	8.2 (1.8,37.0)***	0.8 (0.2,2.9)	0.8 (0.2,3.2)	3.3 (0.7,15.4)	1.7 (1.1–29.9)	3.1 (0.9,10.14
GA at ROM	Aor	1.1 (0.2,6.0)	4.6 (1.9,31.8)***	5.9 (1.9,37.0)*	0.6 (0.1–2.6)	0.2 (0.0,1.7)	1.3 (0.2–11.0)	1.2 (0.18,7.48)
GA at D	Aor	7.2 (1.1,47.6)*	4.3 (1.9,30.9)***	0.7 (0.1,6.2)	0.5 (0.1–2.9)	0.3 (0.0,2.9)	0.9 (0.5–1.8)	7.5 (1.2,48.9)*
LP	Aor	1.1 (0.4,2.9)	2.0 (0.90, 4.6)	1.2 (0.5,2.9)	1.3 (0.5–3.1)	0.5 (0.1,1.9)	0.6 (0.2–11.0)	0.7 (0.3,2.09
BW	Aor	1.9 (0.6–66.9)	1.8 (0.7–5.1)	2.0 (0.9–4.6)	1.5 (0.5–3.9)	0.46 (0.1–1.3)	0.9 (0.3–3.2)	0.2 (0.1,0.8
LD	Aor	2.1 (0.2,22.0)	2.8 (0.43–17.9	0.2 (0.1–0.8)*	1.3 (0.24–6.7)	0.3 (0.0–4.2)	0.3 (0.1–13.5)	0.0 (0.0.01,1.4)

Aor, adjusted odd ratio; NICU, Neonatal intensive care unit; EONS, LP, latency period; LD, labor duration; BW, birth weight; Chor, Chorioamnionitis; Early-onset neonatal sepsis; RDS, respiratory distress syndrome; Stat, statistics; Abs,: antibiotics; GA, Gestational age, and ROM, rupture of membrane; D, delivery.

* & *** indicate statistically significant difference at *p* < 0.01 and *p* < 0.001, respectively.

## Discussion

### Rate of preterm PROM

In this study, the prevalence of preterm PROM was 2.2%, which was in the general range reported globally, 1%–3% (3, 6). The rate in this study was less than in the previous studies (15, 16), while it is slightly higher than in another study conducted before (8).

The discrepancy findings between studies could be attributed to factors such as maternal comorbidities, socioeconomic status of women, and previous obstetric and delivery histories. Previous studies indicated that preterm PROM was attributed to these factors (17–19). Type of pregnancy (single vs. multiple), intrauterine infection, trauma, poor nutrition during pregnancy, vaginal bleeding, educational level, race, sociodemographic factors, genetic predisposition, and uterine over distention were also contributed to preterm PROM (20–23). This indicates that these factors might also contribute to the difference in the rate of preterm PROM between studies. However, the mean age of women, parity, and antenatal care visits in our study and studies conducted before were the same (8, 24), revealing that these factors did not bring the preterm PROM rate difference between the above studies.

### Adverse perinatal outcomes and determinants

Low Apgar score, EONS, RDS, and perinatal mortality were some of the adverse perinatal outcomes assessed in this study. Maternal adverse outcomes such as Chorioamnionitis and placental abruption were also assessed. In this study, determinants for these adverse outcomes were evaluated.

#### Low apgar score at 5th Minute

In this study, among 160 deliveries, neonates with a low Apgar score at the 5th minute were found to be 41 (26%). GA at rupture of membrane and delivery was among the determinant factors for the low Apgar score at the 5th minute. According to the multivariate logistic regression analysis, in this study, the low GA at delivery was the stronger predictor and an important cause for the low Apgar score at the 5th minute. Like the previous study (8), the low GA at rupture of membrane and delivery were the risk factors for the low Apgar score at the 5th minute. However, parity, mode of delivery, use of antibiotics or steroids, maternal age, and latency period were not associated with the low Apgar score at the 5th minute. Unlike our study, other studies showed that low birth weight and maternal age were the determinant factors for the low Apgar score at the 5th minute (25–27). In the study conducted by Getaneh et al. (25), the percentage of women in the advanced age (age >34 years) group was 50.57%, while it was only 6.25% in our study. This variation might contribute to the different findings between these studies. In the study conducted by Zewude et al. (26) and Yeshaneh et al. (27), the amniotic membrane was ruptured at term while it was in the preterm in our study, and this could contribute to the difference in the determinates for the low Apgar score results between these previous and our studies.

According to a study conducted by Dwi and Salan (28), the latency period was also the determinant factor for the low Apgar score at the 5th minute, but not in our study. In addition, socio-demographic, obstetrics, and other differences, variation in the sample size, and latency period classification could contribute to different findings on the association between the latency period and Apgar score.

Besides, although the association was not significant, 50.0% of neonates who had low Apgar scores were from women who had no antenatal care visits, and 25.3% were from women who had antenatal care visits. Yeshaneh et al. (27) showed that antenatal care visits significantly determined the Apgar score at the 5th minute. This indicates that antenatal care packages and visits are essential to adverse perinatal outcomes.

#### NICU admission

Among 133 alive-born neonates, 79 (59%) were admitted to NICU. This result was greater than in the studies conducted before (8, 15). The higher rate of admission to NICU in the current study might be attributed to the increase in the new NICE centers compared to the previous (8). In the study conducted by Sirak and Mesfin (8), only 7.5% of the neonates had a low Apgar score, but 26% had a low Apgar score in our study. Thus, the higher number of neonates with a low Apgar score in our study could be attributed to the higher number of neonates admitted to the NICU. Yet, the rate of EONS has not been passed in the study conducted by Sirac and Mesfin (8); 34.38% of neonates had EONS in our research, which could increase the number of neonates admitted to the NICU. Another study showed that the leading cause of admission was EONS (29). Besides, in the study conducted by (15), women who presented with PPROM that developed Chorioamnionitis were only 4.3%, while 6.88% in our study. Chorioamnionitis increases neonatal adverse outcomes such as RDS, NICU admission, and poor fetal outcomes (29, 30).

In this study, NICU admission was affected by failure to use steroids for lung maturity, early GA at membrane rupture, and delivery and low birth weight. This indicates that the use of antenatal steroids for lung maturation reduces adverse neonatal outcomes. Before, a study indicated that surfactant administration in neonates who received multiple-dose of antenatal corticosteroids was less than in neonates who did not receive steroids (31). Antenatal corticosteroid administration increases lung maturation and reduces neonatal complications such as RDS, NICU admission, and risk of death (32).

Similar to our study, a study conducted by Paudela et al. (11), indicated that one of the risk factors for NICU admission is prelabor rupture of the membrane and preterm births. This indicates that earlier membrane rupture and delivery are the risk factors for NICU admission.

On the other hand, like the previous study (33), low birth weight was also one of the determinant factors for NICU admission in our study. Other studies also indicated that low birth weight was associated with low Apgar scores and neonatal deaths (34, 35). These findings revealed that prematurity and low birth weight are associated with adverse perinatal outcomes. Adverse respiratory outcomes are prevalent among neonates with prematurity and low birth weight (36, 37). Prenatal management in preventing preterm births and low birth weight is essential to reduce adverse perinatal outcomes.

In our study, mode of delivery, latency period, parity, and other variables didn't determine NICU admission. However, a previous study indicated that delivering by cesarean section, being primiparas, and having Chorioamnionitis were the determinants for NICU admission (38). Variations in socio-demographic characteristics, maternal and neonatal conditions, birth weight, birth location, and the neonates' clinical features could be again attributed.

#### EONS, RDS, and perinatal mortality

In this study, adverse perinatal outcomes such as EONS, RDS, and perinatal mortality were higher than in other studies (39, 40). EONS was the most common neonatal morbidity in this study. The rate of EONS (41%) in our study was also higher than in other studies conducted before (40–42). The bivariate analysis showed that earlier GA (<34 weeks) at membrane rupture and delivery was the determinant for EONS, RDS, and perinatal mortality. A study conducted by (43) also showed that earlier GA at amniotic membrane rupture was associated with an increased risk of neonatal sepsis. Similar to the current study, another study also showed that RDS development was attributed to the low GA at membrane rupture (44).

Low birth weight and labor duration were also the determinate factors for the EONS in our study. A review conducted in Ethiopia showed that the risk of developing EONS among newborns with low birth weight and preterm deliveries was significantly higher than in normal neonates (45). Other research articles also showed low birth weight was associated with an increased risk of neonatal sepsis (46, 47). Multivariate logistic regression analysis showed that the strong predictor for the EONS was the GA at membrane rupture than other parameters. This indicates a higher correlation between GA at membrane rupture and EONS.

Though the association was not statistically significant, 58.3% of neonates having EONS were from women who developed Chorioamnionitis. This reveals that amniotic fluid infection can contribute to EONS. Alam et al. (42) reported that Chorioamnionitis was one of the independent risk factors for EONS. A meta-analysis conducted by Chan et al. (48) also indicated that early-life neonatal infection is associated with maternal infection. Another study revealed that Chorioamnionitis is associated with significant maternal and neonatal morbidity and mortality (49). Maternal infection prevention during pregnancy, timely diagnosis, and appropriate treatment is essential to reduce fetal and neonatal adverse outcomes.

Similar to the previous studies (11, 50), the second most common diagnosis among neonates admitted to the NICU was RDS (30%). In addition to the earlier GA membrane rupture and delivery, low birth weight and Chorioamnionitis were the determinate factors for RDS in this study. A study conducted by Landry et al. (51) showed a significant association between RDS and low birth weight. However, another study revealed that birth weight was not a factor for RDS (52).

On the other hand, 50.0% of neonates who had RDS were born from women with Chorioamnionitis and 23.0% of neonates were from women with no Chorioamnionitis. Previous studies indicated that Chorioamnionitis was significantly associated with RDS (30, 53). This shows that maternal infections during pregnancy contribute to adverse neonatal outcomes.

The total fetal deaths before and during labor were 27 (17%), while 6 (5%) neonates died in the first week after delivery. The perinatal mortality rate in this study was 20.63% which was higher than in the studies conducted before (8, 54, 55). The rate of the low Apgar score at the 5th minute in our study was greater than in the study conducted before (8). This could be attributed to the higher perinatal mortality rate in our study. A previous study found an association between a low Apgar score at the 5th minute and neonatal mortality among preterm and term newborns (56). In the study conducted by Mu et al. (55), the association was stronger if delivery was made at earlier GA, revealing that GA at delivery is necessary to determine the adverse perinatal outcomes.

In our study, the low GA at membrane rupture and delivery and birth weight were the determinant factors for perinatal death. Similarly, other studies also indicated that low GA at delivery and birth weight are determinants of perinatal mortality (56–58). In this study, perinatal mortality was not significantly affected by parity, mode of delivery, use of antibiotics and steroids, latency period, RDS, and antenatal care visits. Similarly, a study conducted by Kayiga et al. (59) indicated that the mode of delivery didn't affect perinatal mortality.

However, other studies showed respiratory problems and mode of delivery (55, 60). The disagreement between these studies could be attributed to different factors such as GA at amniotic fluid rupture, delivery, maternal age, and maternal comorbidities during pregnancy, and place of delivery. Previous studies indicated that place of delivery (health center vs. home) and maternal age determined perinatal mortality (25, 61).

#### Clinical chorioamnionitis

Similar to other studies (62, 63), in this study, Chorioamnionitis is one of the adverse outcomes of preterm PROM. Among women with preterm PROM, 7.5% of them had clinical Chorioamnionitis, which is nearly similar to a study conducted before (64). Prolonged latency period and labor duration were significantly associated with clinical amnionitis, indicating that these factors determined these adverse maternal outcomes. However, none of them affected these adverse maternal outcomes during multiple logistic regression analyses. This finding agrees with other studies (39, 65).

Similarly, Sallam (62) showed that the greater the latency period was one of the determinant factors for Chorioamnionitis. Other studies also indicated the presence of an association between the latency period and infection and inflammatory biomarkers (54, 66, 67), and inflammatory biomarkers indicate the presence of Chorioamnionitis (68). Besides, adverse perinatal outcomes were protected against shortened latency (47).

However, another study showed that a prolonged latency period (≥72 h) did not increase maternal morbidities such as Chorioamnionitis (69). The disagreement between these studies could be attributed to variations in the sample size used, GA at delivery, maternal age, maternal social factors, and maternal comorbidities during pregnancy. Maternal age is associated with Chorioamnionitis (70). Cavazos-Rehg et al. (70) reported that maternal age greater than or equal to 35 years was one of the risk factors for Chorioamnionitis. According to another study, maternal comorbidities during pregnancy were attributed to Chorioamnionitis (71).

Similar to another study (72), in this study, GA at membrane rupture and delivery, use of prophylactic antibiotics, and steroids were not significantly associated with the risk of clinical Chorioamnionitis. However, other studies revealed the association between the use of prophylactic antibiotics and the reduction in Chorioamnionitis (23, 49). Type and dose of antibiotics, time and duration of antibiotics administration, maternal body response to antibiotics, maternal lifestyle, and comorbidities during pregnancy could attribute the difference in findings on the effects of antibiotics on Chorioamnionitis between the current and other studies.

#### Abruption placenta

The percentage of women who developed abruption placenta in this study was 12.5% (*n* = 20) which is higher than a study done by Majo (73). The bivariate logistic regression analysis showed that rupture of membrane and delivery at earlier GA were the determinant factors for placental abruption. According to the multiple logistic regression analysis, GA at delivery was the stronger predictor. However, placental abruption was not affected by other factors such as the use of antenatal antibiotics and steroids, educational status, monthly income, maternal age, latency period, labor duration, parity, antenatal care visits, and birth weight. A similar study was reported by Majo et al. (73), except their study didn't find a significant association between placental abruption and GA at delivery. However other studies indicated that low socioeconomic status and maternal age were the determinant factors for placental abruption (74, 75). Hung et al. (76) also reported that women with an age greater than 35 were at the risk of having placental abruption than women under 35 years.

In the study conducted by Ghaheh et al. (75), women aged 35 or above were at risk of placental abruption more than women under 35 years old. In the previous study, women included in the study were at GA after 28 weeks to the preterm, while women at GA after 34 weeks to the preterm were included in our study. On the other hand, in the previous study, the maternal age of 35 years or above was 38.76%, while only 6.25% was in our study. These variations could attribute to the different findings between the current and previous studies.

Unlike the current study, a previous study showed that fewer antenatal care visits and high parity were determinants for placental abruption (77). In our study, the average maternal age among those with placental abruption was 29.1%, while it was 25.8% in our study. On the other hand, in the previous study, 99.1% of women having placental abruption had antenatal care visits, while it was 95% in our study. These variations could contribute to the different findings in these studies.

Regarding the limitation of this study, although the sample size has been increased in this study when compared with the previous study, still the sample size is small. Follow-up of complications in the first seven days of life had challenges. Looking into women who gave birth and had premature PROM for an extended time is important to manage life-threatening maternal, neonatal, and infant complications, however, we were unable to follow up on these women for more than a week which was another limitation of this study.

## Conclusion

GA at membrane rupture and delivery, and unable to use steroids were significant risks for adverse perinatal outcomes among women with preterm PROM. GA at membrane rupture and delivery was the most important predictor of adverse perinatal outcomes. GA at rupture of the membrane was the stronger predictor for neonatal intensive care unit admission and EONS. GA at delivery was the stronger predictor for the low Apgar score at the 5th minute, EONS, and placental abruption.

## Recommendation

Since adverse perinatal outcomes in this study were mainly affected by GA, improving antenatal care, timely interventions, and early referral of high-risk pregnancies to tertiary-level centers are crucial to reducing the risk of adverse perinatal outcomes. Larger scale studies are essential by including other parameters not assessed in this study, such as detailed previous obstetric histories, nutritional status, and psychiatric problems during pregnancy.

## Data Availability

The raw data supporting the conclusions of this article will be made available by the authors, without undue reservation.
